# Impact of Limited Solvent Capacity on Metabolic Rate, Enzyme Activities, and Metabolite Concentrations of *S. cerevisiae* Glycolysis

**DOI:** 10.1371/journal.pcbi.1000195

**Published:** 2008-10-10

**Authors:** Alexei Vazquez, Marcio A. de Menezes, Albert-László Barabási, Zoltan N. Oltvai

**Affiliations:** 1 The Simons Center for Systems Biology, Institute for Advanced Study, Princeton, New Jersey, United States of America; 2 Instituto de Física, Universidade Federal Fluminense, Rio de Janeiro, Brazil; 3 Center for Complex Network Research, and Department of Physics, Biology, and Computer Science, Northeastern University, Boston, Massachusetts, United States of America; 4 Department of Pathology, University of Pittsburgh, Pittsburgh, Pennsylvania, United States of America; University of Virginia, United States of America

## Abstract

The cell's cytoplasm is crowded by its various molecular components, resulting in a limited solvent capacity for the allocation of new proteins, thus constraining various cellular processes such as metabolism. Here we study the impact of the limited solvent capacity constraint on the metabolic rate, enzyme activities, and metabolite concentrations using a computational model of *Saccharomyces cerevisiae* glycolysis as a case study. We show that given the limited solvent capacity constraint, the optimal enzyme activities and the metabolite concentrations necessary to achieve a maximum rate of glycolysis are in agreement with their experimentally measured values. Furthermore, the predicted maximum glycolytic rate determined by the solvent capacity constraint is close to that measured in vivo. These results indicate that the limited solvent capacity is a relevant constraint acting on *S. cerevisiae* at physiological growth conditions, and that a full kinetic model together with the limited solvent capacity constraint can be used to predict both metabolite concentrations and enzyme activities in vivo.

## Author Summary

The concentration of enzymes and metabolites is continuously adjusted in order to achieve specific metabolic demands. It is highly likely that during evolution global metabolic regulation has evolved such as to achieve a given metabolic demand with an optimal use of intracellular resources. However, the size of enzymes and intermediate metabolites is dramatically different. Enzymes are macromolecules that occupy a relatively large amount of space within a cell's crowded cytoplasm, while metabolites are much smaller. This implies that metabolite concentrations are likely to be adjusted to minimize the overall “enzymatic cost” (in terms of space cost). In this work, we explore this hypothesis using *Saccharomyces cerevisiae* glycolysis as a case study. Our results indicate that metabolite concentrations attain optimal values, minimizing the intracellular space occupied by metabolic enzymes. And, at these optimal concentrations, glycolysis achieves the maximum rate given the intracellular volume fraction occupied by glycolysis enzymes. Taken together with previous studies for *Escherichia coli*, our results indicate that macromolecular crowding is a general constraint on cell metabolism.

## Introduction

Understanding an organism's metabolism at a system level and obtaining quantitative predictions for the different metabolic variables requires the identification and modeling of the physicochemical and regulatory constraints that are relevant at physiological growth conditions. Recently, there has been a surge of interest on how macromolecular crowding, i.e., the crowding of the cytoplasm by various molecular components, affects cellular function, including cell metabolism [1],[2].

At the local scale it is well known that molecular crowding affects the rate of biochemical reactions, diffusion, protein folding and protein-protein association/dissociation [2],[3]. More recently, we have shown that macromolecular crowding acts also at a global scale by imposing a limited solvent capacity. Specifically, we have shown that a flux balance modeling framework that incorporates the limited solvent capacity is successful in predicting the maximum growth rate, the sequence of substrate uptake from a complex medium and, to an extent, the changes in intracellular flux rates upon varying growth rate of the bacterium, *Escherichia coli* [4],[5]. Yet, these studies were limited by the absence of a full kinetic model of *E. coli* cell metabolism, hindering our ability to investigate the impact of the solvent capacity constraint on in vivo metabolite concentrations and enzyme activities.

During cellular metabolism the concentration of enzymes and metabolites are continuously adjusted in order to achieve specific metabolic demands. It is highly likely that during evolution global metabolic regulation has evolved such as to achieve a given metabolic demand with an optimal use of intracellular resources. However, the size of enzymes and intermediate metabolites are dramatically different. Enzymes are macromolecules that occupy a relatively large amount of space within a cell's crowded cytoplasm, while metabolites are much smaller. This implies that metabolite concentrations are likely to be adjusted to minimize the overall “enzymatic cost” (in terms of space cost).

Here we study the validity of this hypothesis by focusing on the glycolysis pathway of the yeast, *Saccharomyces cerevisiae*, for which a kinetic model is available. We show that the maximum glycolysis rate determined by the limited solvent capacity is close to the values measured in vivo. Furthermore, the measured concentration of intermediate metabolites and enzyme activities of glycolysis are in agreement with the predicted values necessary to achieve this maximum glycolysis rate. Taken together these results indicate that the limited solvent capacity constraint is relevant for *S. cerevisiae* at physiological conditions. From the modeling point of view, this work demonstrates that a full kinetic model together with the limited solvent capacity constraint can be used to predict not only the metabolite concentrations, but in vivo enzyme activities as well.

## Results

### Limited Solvent Capacity Constraint

The cell's cytoplasm is characterized by a high concentration of macromolecules [1],[2] resulting in a limited solvent capacity for the allocation of metabolic enzymes. More precisely, given that enzyme molecules have a finite molar volume *v_i_* only a finite number of them fit in a given cell volume *V*. Indeed, if *n_i_* is the number of moles of the *i* ^th^ enzyme, then
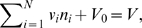
(1)where *V*_0_ accounts for the volume of other cell components and the free volume necessary for cellular transport as well. Equation 1 can be also rewritten as
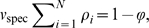
(2)where *ρ_i_* = *n_i_m_i_*/*V* is the enzyme density (enzyme mass/volume), *μ_i_* is the molar mass *v*_spec_ is the specific volume, and *φ* = *V*_0_/*V* is the fraction of cell volume occupied by cell components other than the enzymes catalyzing the reactions of the pathway under consideration, including the free volume necessary for diffusion. The specific volume has been assumed to be constant for all enzymes, an approximation that has been shown to be realistic at least for globular proteins [6]. In this new form we can clearly identify the enzyme density (or mass, given that the volume is constant) as the enzyme associated variable contributing to the solvent capacity constraint. This choice is more appropriate than the enzyme concentration *C_i_* = *n_i_*/*V* (moles/volume) because the specific volume is approximately constant across enzymes, while the molar volume can exhibit significant variations. For example, according to experimental data for several globular proteins [6], the molar volume exhibits a 70% variation while the specific volume is almost constant, with a small 2% variation.

The solvent capacity constraint (Equations 1 and 2) thus imposes a limit to the amount of catalytic units (i.e., enzymes) that can be allocated in the cell cytoplasm. In the following we show that this in turn leads to a constraint for the maximum metabolic rate. The rate of the *i* ^th^ reaction per unit of cell dry weight (mol/time/mass) is given by
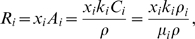
(3)where *A_i_* is the specific enzyme activity, *C_i_* is the enzyme concentration in molar units, *k_i_* is the catalytic constant and *M* is the cell mass. The coefficient *x_i_* is determined by the specific kinetic model: it takes values in the range of 0≤*x_i_*≤1, and it is a function of metabolite concentrations. For example, if the *i* ^th^ reaction is described by Michaelis-Menten kinetics with one substrate then *x_i_* = *S_i_*/(*K_i_*+*S_i_*), where *S_i_* is the substrate concentration and *K_i_* is the equilibrium constant. More generally, *x_i_* is a function of the concentration of substrates, products and other metabolites regulating the enzyme activity. The fact that the reaction rates are proportional to the enzyme densities (Equation 3) suggests that the limited solvent capacity constraint (Equation 2) has an impact on the reaction rates as well. Indeed, from Equations 2 and 3 we obtain
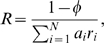
(4)where *R* is the cell metabolic rate (or pathway rate), *r_i_* = *R_i_*/*R* is the rate of reaction *i* relative to the metabolic rate, and
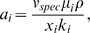
(5)where *ρ* = *M*/*V* is the cell density. We refer to *a_i_* as the crowding coefficients [4],[5], because they quantify the contribution of each reaction rate to molecular crowding. The crowding coefficient of a reaction *i* increases with increasing the enzyme's molar mass *μ_i_* and decreases with increasing catalytic activity *k_i_*. It is also a function of the metabolite concentrations through *x_i_*.

### Hypothetical Three Metabolites Pathway

To illustrate the impact of the limited solvent capacity constraint, we first analyze a hypothetical example, in which we use the relative reaction rates as input parameters, and the metabolite concentrations are the variables to be optimized. Given the reaction rates and the “optimal” metabolite concentrations, the enzyme activities are determined by Equation 3. Finally, the maximum metabolic rate is computed using Equation 4.

**Figure 1:**
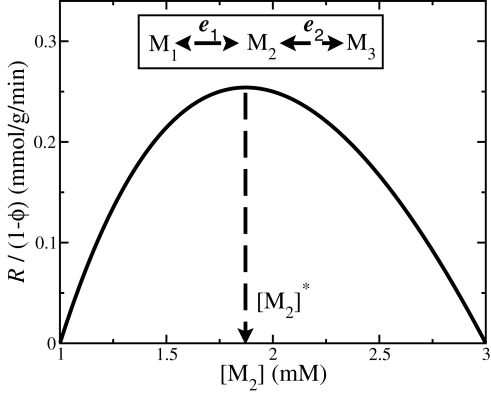
Hypothetical three metabolite pathway. The inset shows a hypothetical three metabolite-containing pathway with two reactions. The main panel displays the pathway rate as a function of the concentration of the intermediate metabolite. Of note, at an intermediate metabolite concentration [M_2_]^*^, the pathway rate achieves a maximum. The plot was obtained using the kinetic parameters indicated in the text.

Consider a metabolic pathway consisting of two reversible reactions converting metabolite M_1_ into M_2_ (reaction 1) and M_2_ into M_3_ (reaction 2), catalyzed by enzymes *e*_1_ and *e*_2_, respectively (Figure 1, inset). The reaction rates per unit of cell mass, *R*_1_ and *R*_2_, are modeled by reversible Michaelis-Menten rate equations, using Equation 3 with
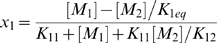
(6)

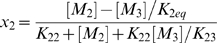
(7)where *K*_1eq_ and *K*_2eq_ are the equilibrium constants of reaction 1 and 2, respectively, *K_im_* is the Michaelis-Menten constant of metabolite *m* in reaction *i*. From Equations 4 to 7 we finally obtain
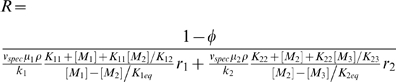
(8)For the purpose of illustration, we assume 1−*ϕ* = 0.01, 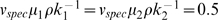 (mmol/h/min)^−1^ (as suggested by typical values reported in [5]), all Michaelis constants equal to 1 mM, and fixed pathway ends metabolite concentrations [M_1_] = 3 mM and [M_2_] = 1 mM. Furthermore, mass conservation for M_2_ implies that *R*_1_ = *R*_2_ = *R* (*r*_1_ = *r*_2_ = 1) in the steady state, where *R* is the pathway rate. When reaction 1 is close to equilibrium [M_2_]≈[M_1_]*K*_1eq_ = 3 mM, the first term in the right hand side becomes very large, resulting in a small pathway rate (Figure 1). When reaction 2 is close to equilibrium [M_2_]≈[M_3_]/*K*_2eq_ = 1 mM, the second term in the right hand side becomes very large, again resulting in a small pathway rate (Figure 1). At an intermediate [M_2_]^*^ between these two extremes the pathway rate achieves its maximum. Therefore, given the solvent capacity constraint, there is an optimal metabolite concentration resulting in a maximum pathway rate.

### *S. cerevisiae* Glycolysis

**Figure 2:**
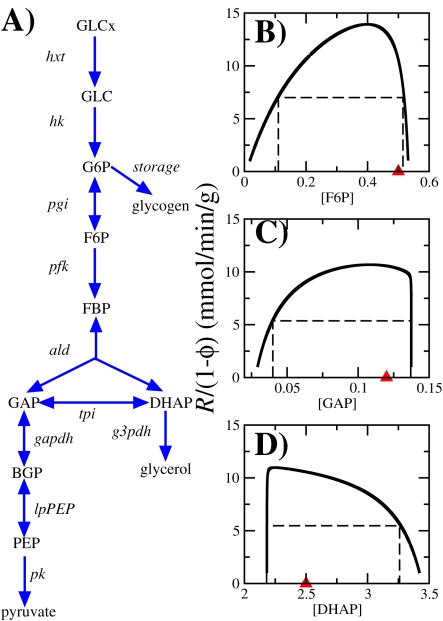
*S. cerevisiae* glycolysis. (A) Schematic representation of glycolysis in *S. cerevisiae*. Metabolites: GLCx, external glucose; GLC, glucose; G6P, glucose 6-phosphate; F6P, fructose 6-phosphate; FBP, fructose 1,6-bisphosphate; DHAP, glycerone phosphate; GAP, D-glyceraldehyde 3-phosphate; BPG, 1,3-bisphosphoglycerate; and PEP, phospho-enol-pyruvate. Reactions: *hxt*, glucose transport; *hk*, hexokinase; *pgi*, phosphogluco isomerase; *pfk*, phospho-fructokinase; *ald*, fructose 1,6-bisphosphate aldolase; *tpi*, triosephosphate isomerase; *gapdh*, D-glyceraldehyde 3-phosphate dehydrogenase; *lpPEP*, reactions from BGP to PEP; *pk*, pyruvate kinase; and *g3pdh*, glycerol 3-phosphate dehydrogenase. (B,C,D) Predicted glycolysis rate as a function of the concentrations of intermediary metabolites in the *S. cerevisiae* glycolysis pathway (in mM). The experimentally determined metabolite levels (from [7]) are indicated by the red triangles. The dashed lines indicate the concentration intervals resulting in 50% or more of the maximum rate.

Next, we investigate whether the observation of an optimal metabolite concentration for maximum pathway rate extrapolates to a more realistic scenario. For this purpose we use the glycolysis pathway of the yeast *S. cerevisiae* (Figure 2A) as a case study. Glycolysis represents a universal pathway for energy production in all domains of life. In *S. cerevisiae* it has been studied extensively resulting in the description of a rate equation model for each of its reactions [7],[8]. In particular, we consider the kinetic model developed in [7] (see Methods). To compare our predictions with experimentally determined values we consider the glycolysis reaction rates and metabolite concentrations reported in [7] and the enzyme activities reported in [8].

In analogy with the three metabolites case study (Figure 1), first we investigate the dependency of the glycolysis rate *R*, represented by the glucose uptake, on the concentration of a given metabolite. In this case we fix all other metabolite concentrations and all relative reaction rates (reaction rate/glycolysis rate) to their experimentally determined values. By doing so the predicted glycolysis rate is an implicit function of the free metabolite concentration alone, through Equation 4. For example, Figure 2B displays the maximum metabolic rate *R* as a function of the concentration of fructose-6-phosphate (F6P). *R* is predicted to achieve a maximum around a F6P concentration of 0.4 mM, close to its experimentally determined value of 0.5 mM [7] (red triangle in Figure 2B). Similar conclusions are obtained for D-glyceraldehyde-3-phosphate (GAP) (Figure 2C) and glycerone-phosphate (DHAP) (Figure 2D). This analysis corroborates that there is an optimal metabolite concentration maximizing *R* and, more importantly, that this concentration is very close to the experimentally determined metabolite concentrations. In all cases the measured metabolite concentrations are within the range of 50% or more of the maximum glycolysis rate.

**Figure 3:**
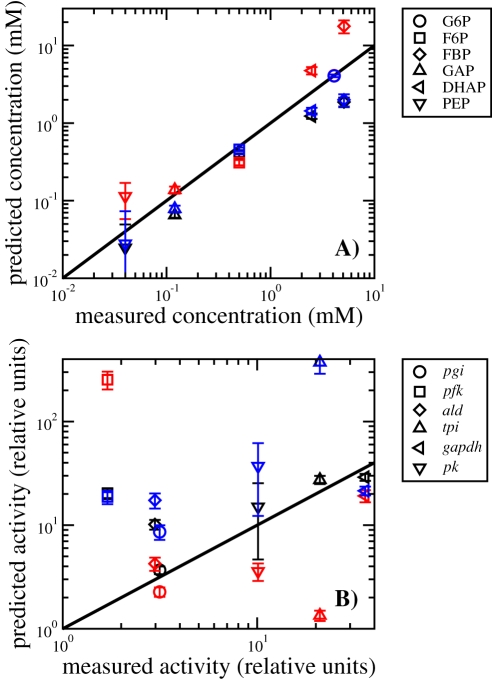
Correlation between predictions vs. experimental data. (A) The predicted metabolite concentrations are plotted as a function of the experimentally determined values (black symbols). The error bars represent the standard deviations, upon generating 100 random sets of kinetic parameters. The solid line corresponds with the coincidence of measured and predicted values, indicating a strong correlation between them. (B) The predicted enzyme activities are plotted as a function of the experimentally determined values, measured in units of the glycolysis rate (black symbols). The error bars represent the standard deviations, upon generating 100 random sets of kinetic parameters. The solid line corresponds with the coincidence of measured and predicted values, indicating a strong correlation between them. In both cases, the red and blue symbols were obtained using the more general optimization objective *R* = (1−*ϕ*)/Σ^*N*^_*i* = 1_ (*a_i_r_i_*)*^H^*, with *H* = 0.1 and 10, respectively.

To further test the optimal metabolite concentration hypothesis, we perform a global optimization and simultaneously compute the optimal concentrations of the glycolysis intermediate metabolites. In this case we fix the concentrations of external glucose and co-factors and all relative reaction rates to their experimentally determined values. By doing so the predicted glycolysis rate is an implicit function of the glycolysis intermediate metabolite concentrations, through Equation 4. The optimal intermediate metabolite concentrations are those maximizing Equation 4. Figure 3A displays the predicted optimal metabolite concentrations as a function of their experimentally determined values (black symbols), the line representing a perfect match. The agreement is remarkably good given the wide range of metabolite concentrations. For phospho-enol-pyruvate (PEP), the predicted value is very sensitive to the model parameters, as indicated by the wide error bars. For fructose 1,6-biphosphate (FBP) the predicted value is smaller by a factor of five than the experimental value, but it is still within range. Taken together, these results indicate that the measured concentrations of intermediate metabolites in the *S. cerevisiae* glycolysis are close to the predicted optimal values maximizing the glycolysis rate given the limited solvent capacity constraint.

Using the optimal intermediate metabolite concentrations we can make predictions for the enzyme activities as well. Indeed, from the first equality in Equation 3 it follows that
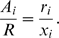
(9)The reaction rates relative to the glycolysis rate *r_i_* are obtained from experimental data, while *x_i_* are obtained after substituting the predicted optimal metabolite concentrations on the reaction's kinetic models. Figure 3B displays the predicted enzyme activities (in units of the glycolysis rate) as a function of the experimentally determined values (black symbols), the line representing a perfect match. In most cases we obtain a relatively good agreement between experimentally measured and predicted values, with the exception of phosphofructokinase (*pfk*), for which the measured enzyme activities are significantly overestimated. Of note, for pyruvate kinase (*pk*) the predictions are significantly affected by the model parameters, as indicated by the wide error bars.

The preceding analysis does not exclude the possibility that other constraints could result in a good agreement as well. To address this point we consider the more general optimization objective *R* = (1−*ϕ*)/Σ^*N*^_*i* = 1_ (*a_i_r_i_*)*^H^*, parametrized by the exponent *H*. Although this objective is not inspired by a biological intuition, it allows us to explore other possibilities beyond the original case *H* = 1. Figure 3 show our predictions for the case *H* = 0.1 (red symbols) and *H* = 10 (blue symbols), representing a milder and a stronger dependency with the crowding coefficients *a_i_*, respectively. For *H* = 0.1, 1.0 and 10 the predicted metabolite concentrations are in good agreement with the experimental values. Furthermore, when we allow sub-optimal metabolite concentrations resulting in a glycolysis rate below it s maximum our predictions are also in the range of the experimental values (see Protocol S1, Table IV). These results indicate that it is sufficient that the optimization objective is a monotonic decreasing function of the crowding coefficients. When the latter is satisfied the metabolite concentrations are up to a great extent constrained by the kinetic model.

This is not, however, the case for the enzyme activities. For *H* = 0.1 and the enzymes *pfk*, *tpi* and *pk*, there is a large deviation from the perfect match line. For *H* = 10 and the enzymes *tpi* and *pk*, there is a large deviation from the perfect match line as well. Overall, *H* = 1 gives the better agreement between enzyme activity predictions and their measured values. In addition, it provides a clear biophysical interpretation of the solvent capacity constraint (*H* = 1).

Finally, we use Equation 4 to compute the maximum glycolysis rate as determined by the limited solvent capacity constraint. The global optimization predicts the glycolysis rate *R* = (1−*ϕ*)×12.5 mmol/min/g dry weight. Taking into account that about 30% [9] of the cell is occupied by cell components excluding water, that proteins account for ∼45% of the dry weight [10], and that of these glycolytic enzymes account for ∼22% [11] of the protein mass we obtain 1−*ϕ*∼0.03. Therefore, given that glycolysis enzymes occupy only 3% of the cell volume, we obtain *R*∼0.38 mmol/min/g dry weight. This prediction is in very good agreement with the experimentally determined glycolysis rate of *S. cerevisiae*, ranging between 0.1 to 0.4 mmol/min/g dry weight [8],[12].

## Discussion

The successful modeling of cell metabolism requires the understanding of the physicochemical constraints that are relevant at physiological growth conditions. In our previous work focusing on *E. coli* we have reported results indicating that the limited solvent capacity is an important constraint on cell metabolism, especially under nutrient-rich growth conditions [4],[5]. Using a flux balance approach that incorporates this constraint we predicted the maximum growth rate in different carbon sources [4], the sequence and mode of substrate uptake and utilization from a complex medium [4], and the changes in intracellular flux rates upon varying *E. coli* cells' growth rate [5]. More importantly, these predictions were in good agreement with experimentally determined values.

Here we have extended the study of the impact of the limited solvent capacity by (i) considering a different organism (*S. cerevisiae*), and (ii) a full kinetic model of glycolysis. Using the full kinetic model of *S. cerevisiae* glycolysis, we have demonstrated that the predicted optimal intermediate metabolite concentrations and enzyme activites are in good agreement with the corresponding experimental values. Discrepancies were only observed in association with two different steps in the glycolysis pathway, namely the reaction catalyzed by *pfk* and the reactions between BPG and PEP. The experimental values measurements from cell extracts [8] and, except for potential experimental caveats, they represent phyiological conditions. We thus we believe that the larger deviations for these enzymes are determined by inconsistencies in the kinetic model equations and/or kinetic model parameters. Finally, the glycolysis rate achieved at the optimal metabolite concentrations is in the range of the experimentally measured values.

From the quantitative modeling point of view our results indicate that a full kinetic model together with the solvent capacity constraint can be used to make predictions for the metabolite concentrations and enzyme activities. Thus, we propose the simultaneous optimization of intermediate metabolite concentrations, maximizing the metabolic rate given the solvent capacity, as a method to computationally predict the concentrations of a metabolic pathway's intermediate metabolites and enzyme activities. We have demonstrated the applicability of this method by computing the concentration of *S. cerevisiae* glycolysis intermediate metabolites, resulting in a good agreement with published data.

The hypothesis that high concentration of macromolecules in the cell's cytoplasm imposes a global constraint on the metabolic capacity of an organism has been studied in the past [13],[14],[15]. In most cases [14],[15] it has been postulated that there is a bound to the total enzyme concentration (moles/volume). Yet, -to our knowledge-, no clear explanation has been provided to support that choice. In contrast, our starting postulate is an undeniable physical constraint, the total cell volume (Equation 1). Under this constraint, the enzyme molar volumes are the primary magnitude quantifying the enzymatic cost. In turn, since the enzyme-specific volumes are approximately constant, we can use the enzyme density (mass/volume) as an alternative measure of enzymatic cost.

This modeling framework has advantages and disadvantages with respect to more traditional approaches based on dynamical systems modeling. As an advantage, our method does not require as input parameters the enzyme activities but rather make quantitative predictions for them. On the other hand, our method is based on a steady-state approximation. Therefore, in its present form, it cannot be used to understand dynamical processes, such as the observed metabolite concentration oscillations in *S. cerevisiae* cells when growing at high glucose concentrations [7].

## Methods

### Kinetic Model of Glycolysis

We use the *S. cerevisiae* glycolysis model reported in [7] (see Protocol S1 for details). The only modification is the extension of the phsophofructokinase (*pfk*) kinetic model from an irreversible to a reversible model.

### Catalytic Constants, Cell Density, Specific Volume

The catalytic constants were obtained from experimental estimates for *Saccharomyces carlsbergensis* [16], except for glycerol 3-phosphate dehydrogenase that was obtained from an estimate for *Eidolon helvum* [17]. For the cell density we use an estimate reported for *E. coli*, *ρ* = 0.34 g/ml [18]. The specific volume was estimated for several proteins using the molar volumes and masses reported in [6], resulting in average of 0.73 ml/g and standard deviation of 0.02 ml/g. See Protocol S1 for details.

### Optimal Metabolite Concentrations

The optimal metabolite concentrations are obtained maximizing Equation 4 with respect to the free metabolite concentrations. In the case of Figure 2B–2D, all metabolite concentrations are fixed to their experimental values, except for the metabolite indicated by the X-axis. In the case of Figure 3A and 3B, all intermediate metabolite concentrations are optimized, keeping fixed the concentration of external glucose and cofactors (ATP, ADP, AMP, NADH, NAD). In both cases the reaction rates relative to the glycolysis rate (*r_i_*) were taken as input parameters, using the values reported in [7]. The maximization was performed using simulated annealing [19].

### Parameter Sensitivity

To analyze the sensitivity of our predictions to the model parameters we have generated random sets of kinetic parameters, assuming a 10% variation of the fixed metabolite concentrations and all kinetic constants except for the catalytic activities. For the latter we assumed a larger variation of 50%, because they were estimated from a different organism. For each set of parameters we make predictions for the metabolite concentrations and enzyme activities. Figure 3 reports the mean values and standard deviations.

## Supporting Information

Protocol S1 Details on the rate equation model used, the utilized model parameters, and the glycolysis rate and optimal metabolite concentrations.
(0.10 MB PDF)
