# Supplementary material for: Impact of Limited Solvent Capacity on Metabolic Rate, Enzyme Activities, and Metabolite Concentrations of S. cerevisiae Glycolysis
Source: PLoS Comput Biol. 2008 Oct 10;4(10):e1000195. doi: 10.1371/journal.pcbi.1000195 (PMC2533405; doi:10.1371/journal.pcbi.1000195)
Supplement: Protocol S1 — Details on the rate equation model used, the utilized model parameters, and the glycolysis rate and optimal metabolite concentrations. (0.10 MB PDF) [file pcbi.1000195.s001.pdf]

Impact of limited solvent capacity on metabolic rate, enzyme activities and metabolite concentrations of *S. cerevisiae* glycolysis
Protocol S1

Alexei Vazquez, Marcio Argollo de Menezes, Albert-László Barabási, and Zoltán N. Oltvai

I. SCHEMATIC REPRESENTATION OF *SACCHAROMYCES CEREVISIAE* GLYCOLYSIS

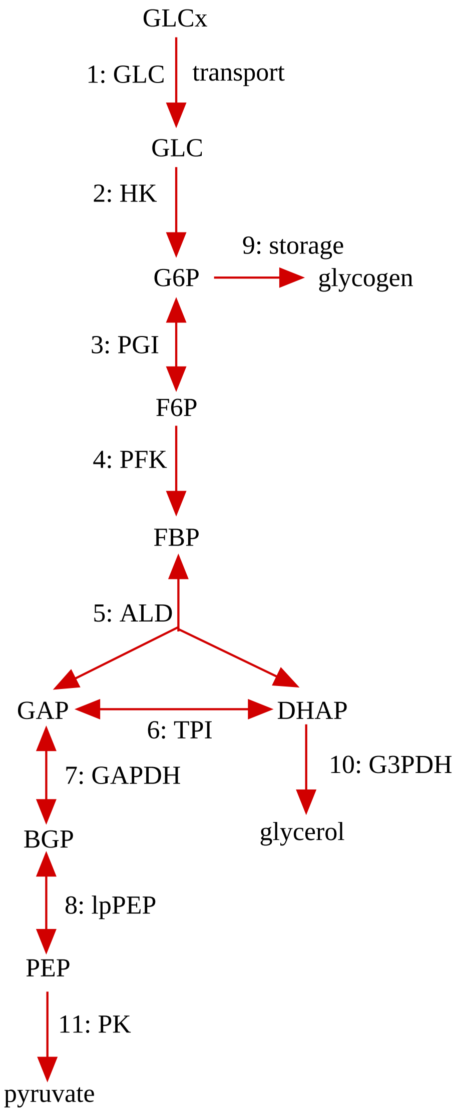

1:	GLCx	$\leftrightarrow$	GLC
2:	GLC + ATP	$\rightarrow$	G6P + ATP
3:	G6P	$\leftrightarrow$	F6P
4:	F6P + ATP	$\leftrightarrow$	FBP + ADP
5:	FBP	$\leftrightarrow$	GAP + DHAP
6:	DHAP	$\leftrightarrow$	GAP
7:	GAP + NAD	$\leftrightarrow$	BGP + NADH
8:	BGP + ADP	$\leftrightarrow$	PEP + ATP
9:	G6P + ATP	$\rightarrow$	ADP
10:	DHAP + NADH	$\rightarrow$	GLYC + NAD
11:	PEP + ADP	$\rightarrow$	PYR + ATP

II. MODEL AND MODEL PARAMETERS

A. Optimization objective

The optimization objective is the glycolysis rate

$$\frac{R}{1-\phi} = \frac{1}{\sum_{i=2}^{10} a_i r_i} \tag{1}$$

where ϕ is the fraction of cell volume occupied by cell components other than glycolysis enzymes, r_i is the rate of the i-th reaction relative to the glycolysis rate,

$$a_i = \frac{v_{spec}\mu_i\rho}{x_i k_i} \tag{2}$$

is the crowding coefficient associated with the i-th reaction, v_{spec} is the specific volume, and μ_i and k_i are the molar mass and catalytic constant of the enzyme catalyzing the i-th reaction. Note that the transport (3) and storage (11) reactions have been excluded. The former does not contribute to the cytoplasm crowding and the latter is considered and step outside glycolysis.

Given the storage rate and the concentration of ATP, the rate equation (13) below determines the concentration of G6P. Furthermore, given this G6P concentration, and the concentration of extracellular glucose, ATP and ADP, the rate equation (3) determines the concentration of intracellular glucose. The remaining metabolite concentrations are obtained such that to maximize (1).

B. Rate equation models, as reported in Ref. [1]

1: Glucose transport (TRANS)

$$v_1 = \frac{1 + \frac{[GLCx]}{K_{1GLC}} + \frac{P_1\frac{[GLCx]}{K_{1GLC}}+1}{P_1\frac{[GLC]}{K_{1GLC}}+1}\left(1 + \frac{[GLC]}{K_{1GLC}} + \frac{[G6P]}{K_{1IGLC}} + \frac{[GLC][G6P]}{K_{1GLC}K_{1IIGLC}}\right)}{\frac{[GLCx]-[GLC]}{K_{1GLC}}} V_{1,max} \tag{3}$$

2: Hexokinase (HK)

$$x_2 = \frac{K_{2DGLC}K_{2ATP} + K_{2GLC}[ATP] + K_{2ATP}[GLC] + [GLC][ATP]}{[ATP][GLC]} \tag{4}$$

3: Phosphoglucoisomerase (PGI)

$$x_3 = \frac{K_{3G6P} + [G6P] + \frac{K_{3G6P}}{K_{3F6P}}[F6P]}{[G6P] - \frac{[F6P]}{K_{3eq}}} \tag{5}$$

4: Phosphofructokinase-1 (PFK)

$$x_4 = \frac{K_{5F6P}\left(1 + \kappa_4\frac{[ATP]^2}{[ADP]^2}\right) + \left([F6P] + \frac{K_{4F6P}}{K_{4FBP}}[FBP]\right)^2}{\left([F6P] - \frac{[FBP]}{K_{4eq}}\right)\left([F6P] + \frac{K_{4F6P}}{K_{4FBP}}[FBP]\right)} \tag{6}$$

This reaction is generally considered as irreversible. Ignoring its reversibility would result, however, in infinitely large values for FBP. Therefore, we have made the reversible extension of this model following [2].

5: Aldolase (ALD)

$$x_5 = \frac{K_{5FBP} + [FBP] + \frac{[GAP]K_{5DHAP}V_{5f}}{K_{5eq}V_{5r}} + \frac{[DHAP]K_{5GAP}V_{5f}}{K_{5eq}V_{5r}} + \frac{[FBP][GAP]}{K_{5IGAP}} + \frac{[GAP][DHAP]V_{5f}}{K_{5eq}V_{5r}}}{[FBP] - \frac{[GAP][DHAP]}{K_{5eq}}} \tag{7}$$

6: Triosephosphate isomerase (TPI)

$$x_6 = \frac{K_{6DHAP} + [DHAP] + \frac{K_{6DHAP}}{K_{6GAP}}[GAP]}{[DHAP] - \frac{[GAP]}{K_{6eq}}} \tag{8}$$

7: Glyceraldehyde 3-phosphate dehydrogenase (GAPDH)

$$x_7 = \frac{K_{7GAP}K_{7NAD}\left(1 + \frac{[GAP]}{K_{7GAP}} + \frac{[BGP]}{K_{7BGP}}\right)\left(1 + \frac{[NAD]}{K_{7NAD}} + \frac{[NADH]}{K_{7NADH}}\right)}{[GAP][NAD] - \frac{[BGP][NADH]}{K_{7eq}}} \tag{9}$$

8: (lpPEP)

$$v_8 = k_{8f}[BGP][ADP] - k_{8r}[PEP][ATP] \tag{10}$$

9: Pyruvate kinase (PK)

$$x_9 = \frac{(K_{9PEP} + [PEP])(K_{9ADP} + [ADP])}{[ADP][PEP]} \tag{11}$$

10: Glycerol 3-phosphate dehydrogenase (G3PDH)

$$x_{10} = \frac{K_{10DHAP}\left(1 + \frac{K_{15INADH}}{[NADH]}\left(1 + \frac{[NAD]}{K_{10INAD}}\right)\right)[DHAP]\left(1 + \frac{K_{15NADH}}{[NADH]}\left(1 + \frac{[NAD]}{K_{10NAD}}\right)\right)}{[DHAP]} \tag{12}$$

11: Storage

$$v_{11} = k_{11}[ATP][G6P] \tag{13}$$

C. Kinetic constants, as reported in Ref. [1]

Reaction	Parameter	Value
1: TRANS	K_{1GLC}	1.7
	K_{1IG6P}	1.2
	K_{1IIG6P}	7.2
	P_1	1
	$V_{1,max}$	1015 mM/min
2: HK	K_{2ATP}	0.1
	K_{3GLC}	0
	K_{3DGLC}	0.37
3: PGI	K_{3G6P}	0.8
	K_{3F6P}	0.15
	K_{3eq}	0.13
4: PFK	K_{4F6P}	0.021
	K_{4FBP}	0.003^a
	κ_4	0.15
	K_{4eq}	800.0^b
5: ALD	V_{5f}/V_{5r}	0.2
	K_{5FBP}	0.3
	K_{5GAP}	4.0
	K_{5DHAP}	2.0
	K_{5IGAP}	10.0
	K_{5eq}	0.081
6: TPI	K_{6DHAP}	1.23
	K_{6GAP}	1.27
	K_{6eq}	0.055
7: GAPDH	K_{7GAP}	0.6
	K_{7BGP}	0.01
	K_{7NAD}	0.1
	K_{7NADH}	0.06
	K_{7eq}	0.0055
8: lpPEP	k_{8f}	443900
	k_{8r}	1529
9: PK	K_{9ADP}	0.17
	K_{9PEP}	0.2
10: G3PDH	K_{10NADH}	0.13
	K_{10DHAP}	25
	$K_{10INADH}$	0.034
	K_{10INAD}	0.13
11: storage	k_{11}	2.26

[a] This parameter was fitted to obtain the best agreement between the measured FBP concentration and the value predicted by the maximization of (1) with all other metabolite concentrations fixed to their experimentally determined values. [b] From Ref. [4].

D. Enzyme molar masses and catalytic constants

Nomenclature	Enzyme	Molar mass (g/mol)	Catalytic constant (1/s)
hk	hexokinase	53738.7	96
pgi	phosphogluco isomerase	61299.5	120
pfk	phospho-fructokinase	107971	376
ald	fructose 1,6-bisphosphate aldolase	39620.9	142
tpi	triosephosphate isomerase	26795.6	3580
gapdh	D-glyceraldehyde 3-phosphate dehydrogenase	35750	144
pk	pyruvate kinase	55195.5	632
g3pdh	glycerol 3-phosphate dehydrogenase	42869.1	33.3

The catalytic constants were obtained from experimental estimates for *Saccharomyces carlsbergensis* [3], except for *g3pdh* that was obtained from an estimate for *Edidolon helvum* [5].

E. Specific volume and cell density

Parameter	Name	Value	Source
v_{spec}	Specific volume	0.73 ml/g	globular proteins [6]
ρ	Cell density	0.34 g/ml	*E. coli* [7]

F. Reaction rates, as reported in Ref. [1]

Reaction/pathway	nomenclature	relative rate
glycolysis	v_0	27mM/min
fermentation	r_{ferm}	0.12
glycerol production	r_{glyc}	0.13
lactonitrile formation	r_{lact}	0.04
glycogen buildup	r_{stor}	0.71
HK	r_2	$r_{ferm} + r_{glyc} + r_{lact} + 2r_{stor}$
PGI	r_3	$r_{ferm} + r_{glyc} + r_{lact} + r_{stor}$
PFK	r_4	$r_{ferm} + r_{glyc} + r_{lact} + r_{stor}$
ALD	r_5	$r_{ferm} + r_{glyc} + r_{lact} + r_{stor}$
TPI	r_6	$r_{ferm} + r_{stor}$
GAPDH	r_7	$2r_{ferm} + r_{glyc} + r_{lact} + 2r_{stor}$
lpPEP	r_8	$2r_{ferm} + r_{glyc} + r_{lact} + 2r_{stor}$
PK	r_9	$2r_{ferm} + r_{glyc} + r_{lact} + 2r_{stor}$
G3PDH	r_{10}	$r_{glyc} + r_{lact}$

G. Fixed metabolite concentrations, as reported in Ref. [1]

Nomenclature	Metabolite	Experiment (mM)
GLCx	External glucose	1.6
ATP	Adenosine 5'-triphosphate	2.1
ADP	Adenosine 5'-biphosphate	1.5
AMP	Adenosine 5'-monophosphate	0.33
NADH	Nicotinamide adenine dinucleotide (reduced form)	0.33
NAD	Nicotinamide adenine dinucleotide (oxidized form)	0.65

III. EXPERIMENTAL DATA USED IN THE COMPARISON WITH THE THEORETICAL PREDICTIONS

A. Metabolite concentrations, as reported in Ref. [1]

Nomenclature	Metabolite	Concentration (mM)
G6P	Glucose 6-phosphate	4.1
F6P	Fructose 6-phosphate	0.5
FBP	Fructose 1,6-biphosphate	5.1
GAP	Glyceraldehyde 3-phosphate	0.12
DHAP	Dihydroxyacetone phosphate	2.5
PEP	Phosphoenol pyruvate	0.04

B. Enzyme activities A relative to the glycolysis rate R, as reported in [4]

Nomenclature	Enzyme	Activity (A/R)
pgi	phosphogluco isomerase	3.15
pfk	phospho-fructokinase	1.7
ald	fructose 1,6-bisphosphate aldolase	2.98
tpi	triosephosphate isomerase	21.0
gapdh	D-glyceraldehyde 3-phosphate dehydrogenase	11.0-60.0[a]
pk	pyruvate kinase	10.1

[a] For the forward and reverse reaction. In this case we used the average, 35.5, to make the comparison with the theoretical predictions.

IV. SUBOPTIMAL METABOLITE CONCENTRATIONS

Metabolite	Experimen	Mean	SD
g6p	4.1	4.0	fixed
f6p	0.5	0.40	0.031
fbp	5.1	5.6	10
gap	0.12	0.12	0.013
dhap	2.5	2.5	0.11
bgp	-	0.00033	0.00012
pep	0.04	0.063	0.046

This table shows the mean and standard deviation of metabolite concentrations for suboptimal glycolysis rates. We obtain these values from a Montecarlo sampling of the metabolite concentrations and weight the averages by the exponential factor $e^{\beta R}$. where R is the glycolysis rate (which depends on metabolite concentrations) and β is a control parameter. The latter weight is motivated by the fact that in an exponentially growing culture the cells abundance is proportional to exp(growth-rate$\times$time) and the growth-rate is proportional to the glycolysis rate. We fix a value of β resulting in an R standard deviation of 10value we obtain the mean metabolite concentrations and standard deviations reported in the Supp. Table E. The mean of these suboptimal metabolite concentrations is in the range of the experimental values. And the standard deviations are of the order or smaller than the means. We add some text in the S. cerevisiae glycolysis section and the Supp. Table E addressing these observations.

[1] Hynne F, Dano S, and Sorensen P G (2001) Full-scale model of glycolysis in *Saccharomyces cerevisiae*, Biophys Chem 94: 121-163

[2] Hofmeyr J-H and Cornish-Bowden A (1997) The reversible Hill equation: how to incorporate cooperative enzymes into metabolic models. Compt Appl Biosci 13: 377-385.

[3] Boiteux A and Hess B. (1981) Design of glycolysis, Phisl Trans R Soc Lond B 293: 5-22.

[4] Teusink B et al (2000) Can yeast glycolysis be understood in terms of *in vitro* kinetics of the constituent enzymes? testing biochemistry, Eur J Biochem 267: 5113-5329.

[5] Schomburg I, Chang A, Schomburg D (2002) BRENDA, enzyme data and metabolic information. Nucleic Acids Res 30: 47-49.

[6] Lee B (1983) Calculation of volume fluctuation for globular protein models. Proc Natl Acad Sci U S A 80: 622-626.

[7] Zimmerman SB, Trach SO (1991) Estimation of macromolecule concentrations and excluded volume effects for the cytoplasm of Escherichia coli. J Mol Biol 222: 599620.
